# Fractalkine-CX3CR1 signaling is critical for progesterone-mediated neuroprotection in the retina

**DOI:** 10.1038/srep43067

**Published:** 2017-02-20

**Authors:** Sarah L. Roche, Alice C. Wyse-Jackson, Ana M. Ruiz-Lopez, Ashleigh M. Byrne, Thomas G. Cotter

**Affiliations:** 1 Cell Development and Disease Laboratory, Biochemistry Department, Biosciences Institute, University College Cork, Cork, Ireland

## Abstract

Retinitis pigmentosa (RP) encompasses a group of retinal diseases resulting in photoreceptor loss and blindness. We have previously shown in the rd10 mouse model of RP, that rd10 microglia drive degeneration of viable neurons. Norgestrel, a progesterone analogue, primes viable neurons against potential microglial damage. In the current study we wished to investigate this neuroprotective effect further. We were particularly interested in the role of fractalkine-CX3CR1 signaling, previously shown to mediate photoreceptor-microglia crosstalk and promote survival in the rd10 retina. Norgestrel upregulates fractalkine-CX3CR1 signaling in the rd10 retina, coinciding with photoreceptor survival. We show that Norgestrel-treated photoreceptor-like cells, 661Ws, and C57 explants modulate rd10 microglial activity in co-culture, resulting in increased photoreceptor survival. Assessment of Norgestrel’s neuroprotective effects when fractalkine was knocked-down in 661 W cells and release of fractalkine was reduced in rd10 explants confirms a crucial role for fractalkine-CX3CR1 signaling in Norgestrel-mediated neuroprotection. To further understand the role of fractalkine in neuroprotection, we assessed the release of 40 cytokines in fractalkine-treated rd10 microglia and explants. In both cases, treatment with fractalkine reduced a variety of pro-inflammatory cytokines. These findings further our understanding of Norgestrel’s neuroprotective properties, capable of modulating harmful microglial activity indirectly through photoreceptors, leading to increased neuroprotection.

Retinitis pigmentosa (RP) encompasses a set of hereditary diseases resulting in a progressive loss of rod and subsequently cone photoreceptors, leading to eventual blindness. The rd10 mouse model of RP harbors a mutation in *phosphodiesterase-6b (pde6b*) and is widely used to study retinal degeneration and investigate potential therapeutics for RP. Although microglia are essential in the clearance of cell debris during degeneration in the CNS^1^,^2^,^3^, recent publications have suggested a detrimental role for microglia in the retina, as drivers of retinal degeneration^4^,^5^,^6^,^7^,^8^. Previous studies therefore propose that microglia are not merely bystander cells during retinal degeneration but are actively contributing to disease progression. Hence, microglia represent a potential therapeutic target for the treatment of retinal degeneration. Indeed, genetic and pharmaceutical targeting of microglial activity in the diseased retina is effective in promoting photoreceptor cell survival^5^,^6^,^7^,^9^,^10^,^11^.

Our group has previously reported on the neuroprotective properties of Norgestrel in the retina^7^,^12^,^13^,^14^,^15^,^16^. In such studies we have used a photoreceptor-like cell line, 661 W, to study the stress response of photoreceptors and reveal the signaling pathways leading to Norgestrel-mediated neuroprotection^7^,^13^,^14^,^17^. We have previously shown that isolated rd10 microglia drive degeneration of 661 W cells *in vitro* and that pre-treating 661 W cells with Norgestrel alleviates microglial-driven degeneration^7^. Thus, Norgestrel revealed a principal aspect of its neuroprotective properties; through the modulation of photoreceptor-microglia crosstalk.

Fractalkine (CX3CL1) is a chemokine synthesized as a 50–75 kDa protein^18^. It is glycosylated forming a transmembrane 100 kDa protein^18^,^19^,^20^. Membrane-bound fractalkine consists of a chemokine domain with CX3 C motif, a highly-glycosylated mucin-like stalk, a transmembrane domain and a short cytoplasmic domain^21^. Membrane-bound fractalkine is cleaved by endogenous metalloproteinases, predominantly ADAM10, to release soluble fractalkine (85 kDa)^22^. Fractalkine can be recycled from the membrane and stored in intracellular vesicles^19^,^20^. In the retina, fractalkine’s sole receptor, CX3CR1, is present on microglia^23^. Fractalkine-CX3CR1 signaling provides a means of intercellular signaling between neurons and microglia in the retina.

In the rd10 mouse, we found that Norgestrel upregulated fractalkine-CX3CR1 signaling 1000 fold at the RNA level, during significant protection of photoreceptors^7^. Studies have documented a neuroprotective role for fractalkine-CX3CR1 signaling in the rd10 retina. Although fractalkine is constitutively expressed in the retina throughout postnatal development^19^, retinal development is unaffected by the absence of fractalkine-CX3CR1 signaling^24^. However, with the onset of stressful stimuli, such as downstream effects of the mutation in the rd10 retina, absence of fractalkine signaling results in increased microglial infiltration and phagocytosis of photoreceptors, potentiating disease progression^6^,^9^. Intra-vitreal injection of recombinant fractalkine reduces microglial infiltration, phagocytosis and photoreceptor cell death in the rd10 retina^9^. Previous work thus hints at the involvement of fractalkine-CX3CR1 signaling in Norgestrel-mediated neuroprotection. In the current study, we investigated the role of fractalkine-CX3CR1 signaling in Norgestrel-dependent neuroprotection.

Using a co-culture of C57 explants and rd10 microglia we expand on previous observations to show that rd10 microglia drive degeneration of viable photoreceptors *ex vivo*, as well as 661 W cells *in vitro*. Photoreceptor cell death is abrogated by pre-treating 661 W cells and explants with Norgestrel. Norgestrel-mediated protection is accompanied by less microglial association with 661 W cells and photoreceptors *in vitro* and *ex vivo*. Hypothesizing that fractalkine-CX3CR1 signaling plays a crucial role, we show that fractalkine is upregulated in 661 W cells and C57 explants with Norgestrel. Knockdown of fractalkine in 661 W cells by siRNA confirms that Norgestrel utilizes fractalkine-CX3CR1 signaling to protect 661 W cells from microglial-derived damage. Using an inhibitor of ADAM10 to manipulate the cleavage of fractalkine, we confirm in rd10 explants that the release of soluble fractalkine is critical to Norgestrel-dependent neuroprotection. In addition to induction of a migratory phenotype, we show that soluble fractalkine modulates cytokine release in isolated rd10 microglia and rd10 explants. Taken together, these findings highlight a critical role for fractalkine-CX3CR1 signaling in Norgestrel-dependent neuroprotection and further our understanding of the role of fractalkine in regulating microglial activity in the retina.

## Results

### Norgestrel primes viable cells against potential microglial-derived toxicity

**Figure 1:**
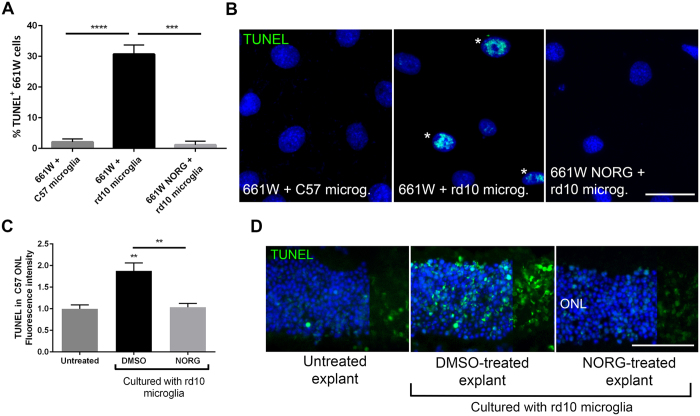
Norgestrel primes viable cells against microglial-derived toxicity. (**A**) Quantification of TUNEL+ 661 W cells pre-treated with 20 μM Norgestrel or vehicle (DMSO) and in co-culture with C57 or rd10 microglia (N = 8 mice for primary culture, n = 6 technical replicates). (**B**) Example images of TUNEL+ 661 W cells (green) in co-culture with microglia. Scale bar 30 μm. (**C**) Quantification of TUNEL fluorescence intensity in P20 C57 explants treated with Norgestrel or vehicle and in co-culture with rd10 microglia (N = 3 explants, n = 4 technical replicates). (**D**) Example images of TUNEL reactivity (green) in the ONL of P20 C57 explants treated with Norgestrel or vehicle and in co-culture with rd10 microglia. Scale bar 50 μm. Hoechst reveals cell nuclei. Results are presented as mean ± SEM (t-test, **p < 0.01, ****p < 0.0001).

As previously described^7^, co-culturing 661 W cells with P15 rd10 microglia for 24hr resulted in a significant increase in 661 W cell death compared to co-culturing with C57 microglia, as assessed by TUNEL (Fig. 1A,B). As expected, pre-treating 661 W cells with 20 μM Norgestrel for 24 hr before co-culture, significantly reduced microglial-driven degeneration^7^ (Fig. 1A,B). In order to determine if photoreceptors responded in a similar way in the retina, we co-cultured P20 C57 retinal explants with rd10 microglia for 19hr. Similar to our observations with 661 W cells, we show that rd10 microglia kill viable photoreceptors *ex vivo* (Fig. 1C,D). Pre-treating C57 explants with 20 μM Norgestrel for 5 hr prior to co-culture with rd10 microglia, significantly reduced microglial-driven degeneration (Fig. 1C,D).

### Norgestrel-treated photoreceptors modulate microglial migration

**Figure 2:**
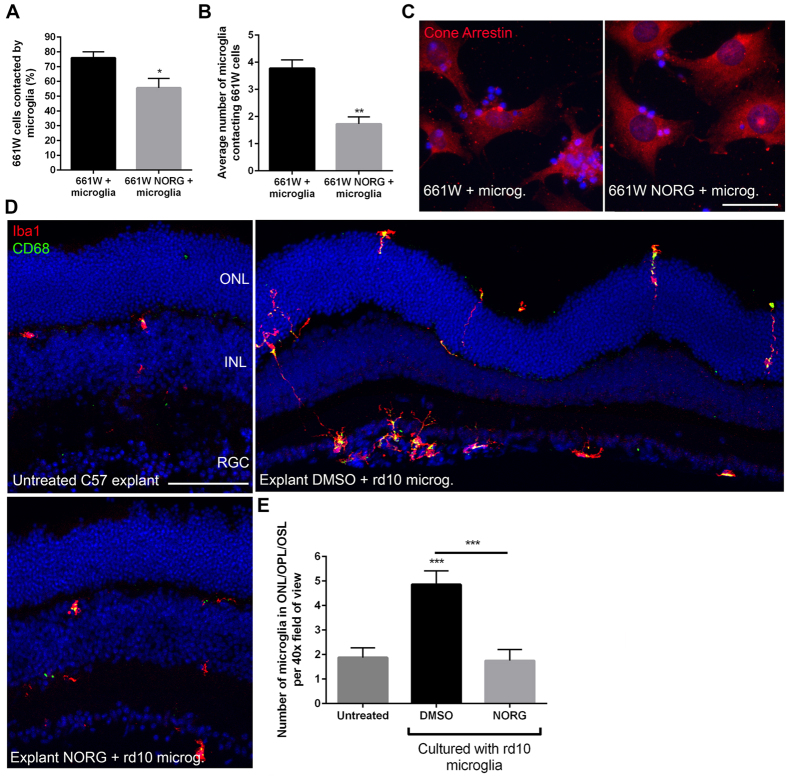
Norgestrel-treated 661 W cells and C57 photoreceptors modulate microglial migration. (**A**) Quantification of the number of 661 W cells pre-treated with 20 μM Norgestrel or vehicle (DMSO) contacted by rd10 microglia. (**B**) Quantification of the average number of microglia contacting 661 W cells in (**A**) (N = 8 mice for primary culture, n = 6 technical replicates). (**C**) Example images of 661 W cells (Cone Arrestin; red) pre-treated with Norgestrel or vehicle in co-culture with rd10 microglia. Scale bar 30 μm. (**D**) Example images of microglia (Iba1; red) and activated microglia (CD68; green) in untreated P20 C57 explants, and explants treated with Norgestrel or vehicle and in co-culture with rd10 microglia. Scale bar 50 μm. (**E**) Quantification of the number of microglia situated in the outer plexiform layer (OPL), outer nuclear layer (ONL) and outer segment layer (OSL) collectively, in untreated P20 C57 explants, and explants treated with Norgestrel or vehicle and in co-culture with rd10 microglia (N = 3 explants, n = 4 technical replicates). Hoechst reveals cell nuclei. Results are presented as mean ± SEM (t-test, *p < 0.05, **p < 0.01, ***p < 0.005).

We wished to understand Norgestrel’s neuroprotective mechanism regulating photoreceptor-microglial crosstalk. Firstly, by quantifying the number of 661 W cells in direct contact with rd10 microglia in co-culture, we show that significantly less 661Ws were contacted by microglia when pre-treated with 20 μM Norgestrel for 24 hr (Fig. 2A,C). The average number of microglia contacting 661 W cells was also significantly less in the Norgestrel-treated group (Fig. 2B,C). As expected in an untreated C57 P20 explant^25^, we observed microglia situated in the outer plexiform layer (OPL) but not in the outer nuclear layer (ONL) (Fig. 2D,E). In the DMSO-treated C57 explant cultured with rd10 microglia, there was a significant increase in the number of microglia in the ONL, OPL and outer segment layer (OSL) collectively (Fig. 2E). Microglia could be seen infiltrating the retina from both the outer and inner retinal surfaces (Fig. 2D; explant DMSO + rd10 microg.). When C57 explants were pre-treated with Norgestrel prior to co-culture with rd10 microglia, there was a significant decrease in the number of microglia situated in the OPL, ONL and OSL (Fig. 2D,E). We therefore hypothesized that Norgestrel was modulating microglial activity indirectly through photoreceptors, by altering the release of chemotactic cytokines from photoreceptors, destined for microglia.

### Norgestrel upregulates fractalkine *in vivo* and *ex vivo*

Fractalkine is a chemotactic cytokine expressed by neurons. In the retina, its sole receptor CX3CR1 is found on microglia^23^ providing an intercellular signaling mechanism between photoreceptors and microglia. We have previously shown that Norgestrel upregulates fractalkine-CX3CR1 signaling in rd10 mice during a time of significant preservation of the ONL^7^. It has also been shown that fractalkine-CX3CR1 signaling is neuroprotective in the rd10 retina^6^,^9^. Based on these findings we hypothesized that Norgestrel was modulating microglial activity indirectly through an upregulation of fractalkine signaling from photoreceptors, consequently providing neuroprotection. To test this hypothesis, we firstly assessed changes in fractalkine levels in cells treated with Norgestrel.

**Figure 3:**
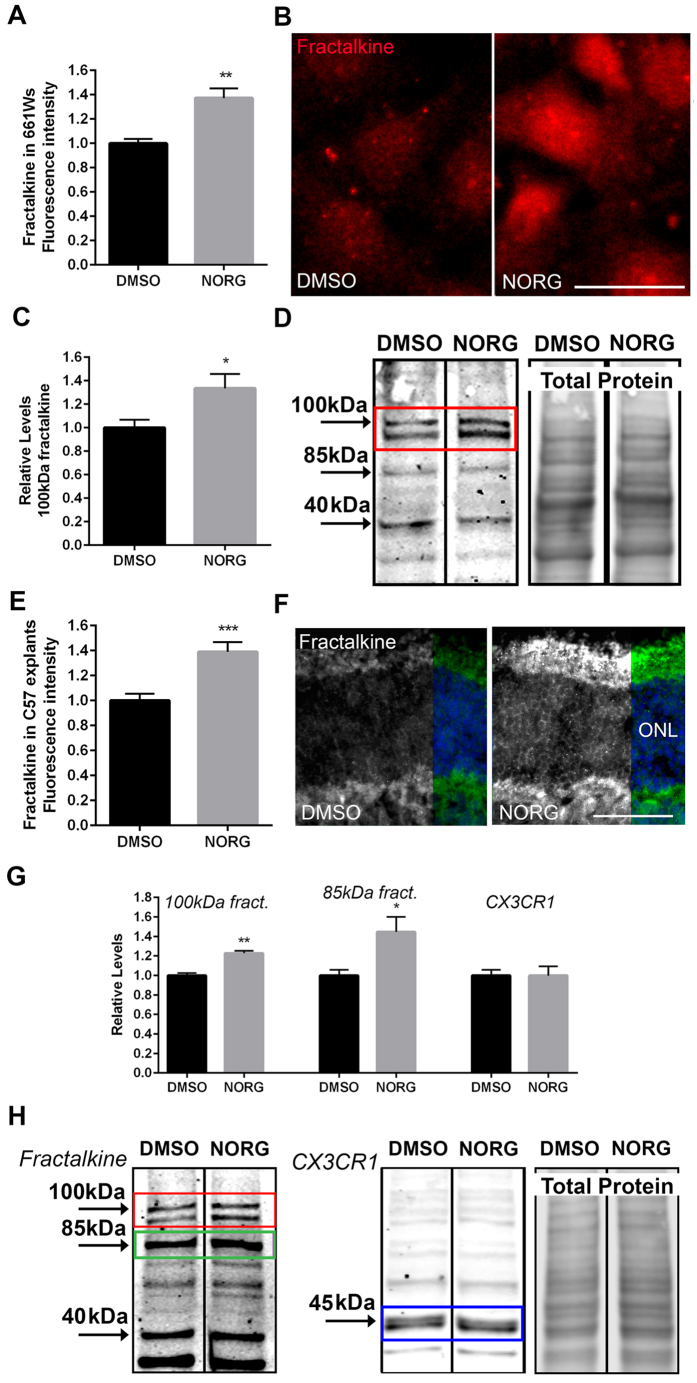
Norgestrel upregulates fractalkine in 661 W cells and C57 explants. (**A**) Quantification of fractalkine fluorescence intensity in 661 W cells treated with 20 μM Norgestrel or vehicle (DMSO) (N = 6 technical replicates). (**B**) Example images of fractalkine immunofluorescence (red) in 661 W cells treated with Norgestrel or vehicle. Scale bar 30 μm. (**C**) Densitometry analysis of Western blots for 100 kDa fractalkine in 661 W cells treated with 20 μM Norgestrel or vehicle (N = 3 biological replicates). (**D**) Western blot for fractalkine in 661 W cells following Norgestrel treatment. Membrane-bound fractalkine at 100 kDa is highlighted (red box). Total protein level is shown alongside. (**E**) Quantification of fractalkine fluorescence intensity in the ONL of P20 C57 explants cells treated with Norgestrel or vehicle (N = 3 explants, n = 4 technical replicates). (**F**) Example images of fractalkine immunofluorescence (green) in the ONL of P20 C57 explants treated with Norgestrel or vehicle. Scale bar 50 μm. (**G**) Densitometry analysis of Western blots for 100 kDa and 85 kDa fractalkine and CX3CR1 in C57 explants cells treated with 20 μM Norgestrel or vehicle (N = 4 explants). (**H**) Western blots for fractalkine and CX3CR1 in P20 C57 explants following Norgestrel treatment. Membrane-bound is observed at 100 kDa (red box) and soluble fractalkine at 85 kDa (green box) and CX3CR1 at 45 kDa (blue box). Total protein level is shown alongside. Hoechst reveals cell nuclei. Results are presented as mean ± SEM (t-test, *p < 0.05, **p < 0.01, ***p < 0.005).

661 W cells treated with 20 μM Norgestrel for 24 hr showed a significant increase in fractalkine as measured by immunofluorescence (Fig. 3A,B). Western blotting confirmed an increase in membrane-bound fractalkine (Fig. 3C,D; 100 kDa). Soluble fractalkine was not observed by Western blot as this would have been released in to the media. Treatment of C57 P20 explants with 20 μM Norgestrel over 5 hr also revealed a significant increase in fractalkine by immunofluorescence (Fig. 3E,F). Both membrane-bound and soluble fractalkine were upregulated as determined by Western blotting (Fig. 3G,H; 100 kDa and 85 kDa). An increase in a band at 95 kDa was also observed with Norgestrel treatment in 661 W cells and C57 explants. This band likely represents an intra-vesicular store of fractalkine that is destined for or has been recycled from the membrane^19^,^20^. Analysis of CX3CR1 by Western blot revealed no change following treatment with Norgestrel (Fig. 3G,H; blue box). These results suggest that Norgestrel utilizes fractalkine-CX3CR1 signaling to protect viable photoreceptors against potential microglial damage.

### Fractalkine signaling is required for Norgestrel-mediated neuroprotection

**Figure 4:**
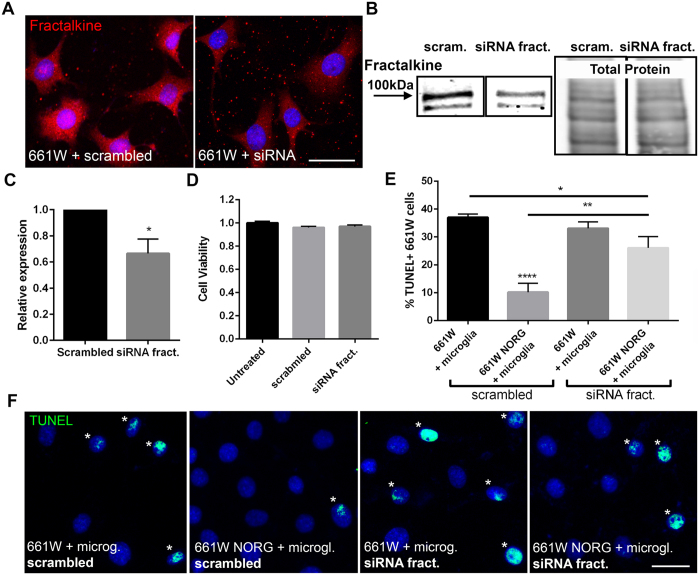
Fractalkine is required for Norgestrel’s neuroprotective effects against rd10 microglia *in vitro*. (**A**) Example images of fractalkine immunofluorescence (red) in 661 W cells treated with siRNA against fractalkine. Scale bar 30 μm. (**B**) Western blot showing decreased levels of fractalkine in 661 W cells treated with siRNA fract. vs scrambled. Total protein level is shown alongside. (**C**) Detection of fractalkine mRNA levels by RT-PCR in 661 W cells following treatment with scrambled or siRNA targeted to fractalkine. (**D**) Cell viability of 661 W cells following siRNA fract. treatment as assessed by the MTS assay (N = 10 technical replicates). (**E**) Quantification of TUNEL+ 661 W cells pre-treated with a combination of scrambled RNA, siRNA fract., 20 μM Norgestrel or vehicle (DMSO) and in co-culture with rd10 microglia (N = 8 mice for primary culture, n = 6 technical replicates). (**F**) Example images of TUNEL+ 661 W cells (green) pre-treated with a combination of scrambled RNA, siRNA fract., Norgestrel or vehicle and in co-culture with rd10 microglia. Scale bar 30 μm. Hoechst reveals cell nuclei. Results are presented as mean ± SEM (two-way ANOVA, *p < 0.05, **p < 0.01, ****p < 0.0001).

**Figure 5:**
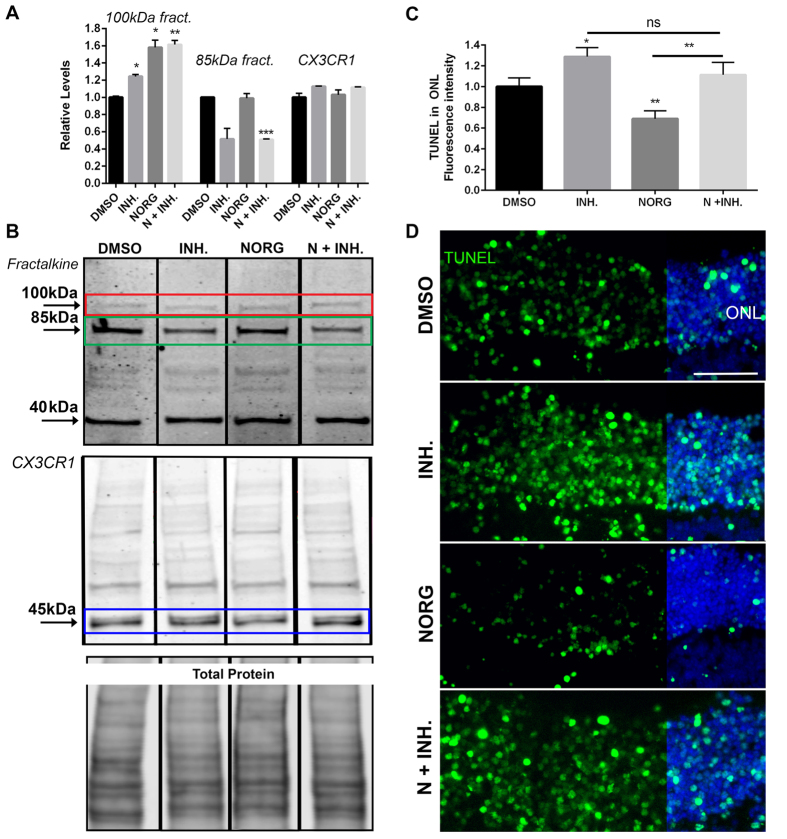
Fractalkine is required for Norgestrel’s neuroprotective effects against rd10 microglia *ex vivo*. (**A**) Densitometry analysis of Western blots for 100 kDa and 85 kDa fractalkine and CX3CR1 in rd10 explants treated with vehicle (DMSO), 100 nM GI254023X (Inhibitor/INH.), 20 μM Norgestrel (NORG) or Norgestrel + GI254023X (N + INH.) (N = 3 explants). (**B**) Western blots showing reduced levels of soluble fractalkine (85 kDa; green box) in the P15 rd10 retina when cleavage is inhibited with 100 nM GI254023X (INH.). Levels of CX3CR1 were unaffected. Total protein level is shown below. (**C**) Quantification of TUNEL fluorescence intensity in P15 rd10 explants treated with vehicle (DMSO), 100 nM GI254023X (Inhibitor/INH.), 20 μM Norgestrel (NORG) or Norgestrel + GI254023X (N + INH.) (N = 3 explants, n = 4 technical replicates). (**D**) Example images of TUNEL reactivity (green) in the ONL of rd10 explants represented in (**C**). Scale bar 30 μm. Hoechst reveals cell nuclei. Results are presented as mean ± SEM (t-test, *p < 0.05, **p < 0.01, p*** < 0.005).

In order to determine if fractalkine-CX3CR1 signaling is essential for Norgestrel’s neuroprotective effects, we targeted fractalkine expression with siRNA in 661 W cells. Knockdown of fractalkine was achieved over 72 hr as evidenced by a substantial loss at the protein level (Fig. 4A,B) and RNA level (Fig. 4C). Importantly, neither transfection with scrambled RNA nor fractalkine siRNA affected 661 W cell viability as compared to untreated 661 W cells (Fig. 4D). 661 W cells treated with scrambled or siRNA fractalkine over 72 hr were subsequently treated with 20 μM Norgestrel or vehicle for 24 hr and co-cultured with rd10 microglia for a further 24 hr. As expected, Norgestrel-treated 661 W cells were significantly protected against microglial damage as assessed by TUNEL (Fig. 4E,F; scrambled). When fractalkine levels were reduced with targeted siRNA, Norgestrel’s protective effects were abrogated (Fig. 4E,F; scrambled NORG vs siRNA fract. NORG). We next sought to evaluate the contribution of fractalkine-CX3CR1 signaling to Norgestrel’s neuroprotective effects in the rd10 retina. As described in previous sections, membrane-bound fractalkine is cleaved to form soluble fractalkine that is released from the cell membrane. ADAM10 has been described as the metalloproteinase largely responsible for this cleavage^22^,^26^. Using a potent inhibitor of ADAM10, GI254023X, we assessed Norgestrel’s neuroprotective effects *ex vivo* when fractalkine cleavage was reduced. GI254023X (Inhibitor/INH.) was effective in reducing levels of soluble fractalkine in rd10 P15 retinal explants over 24 hr (Fig. 5A,B; DMSO vs INH 85 kDa, green box). Treatment of explants with Norgestrel resulted in an increase in membrane-bound fractalkine (Fig. 5A,B; 100 kDa, red box). Levels of CX3CR1, the receptor for fractalkine, remained unchanged following treatment with Norgestrel and/or ADAM10 inhibitor (Fig. 5A,B; blue box). We show that inhibiting the cleavage of fractalkine in rd10 P15 explants results in exacerbated photoreceptor cell death; indicated by a 29% increase in TUNEL compared to vehicle (Fig. 5C,D). This supports previous findings of a neuroprotective role for fractalkine signaling in the rd10 retina^6^,^9^. As expected, Norgestrel provided significant protection to rd10 retinal explants, as evident by a 30% decrease in TUNEL compared to DMSO (Fig. 5C,D). However, when fractalkine cleavage was inhibited in the presence of Norgestrel, TUNEL increased and was similar to levels observed with inhibitor alone (Fig. 5C,D). Norgestrel therefore requires fractalkine-CX3CR1 signaling to generate its neuroprotective effects in the retina.

### Soluble fractalkine induces a migratory phenotype and reduces inflammatory cytokine production in rd10 microglia

**Figure 6:**
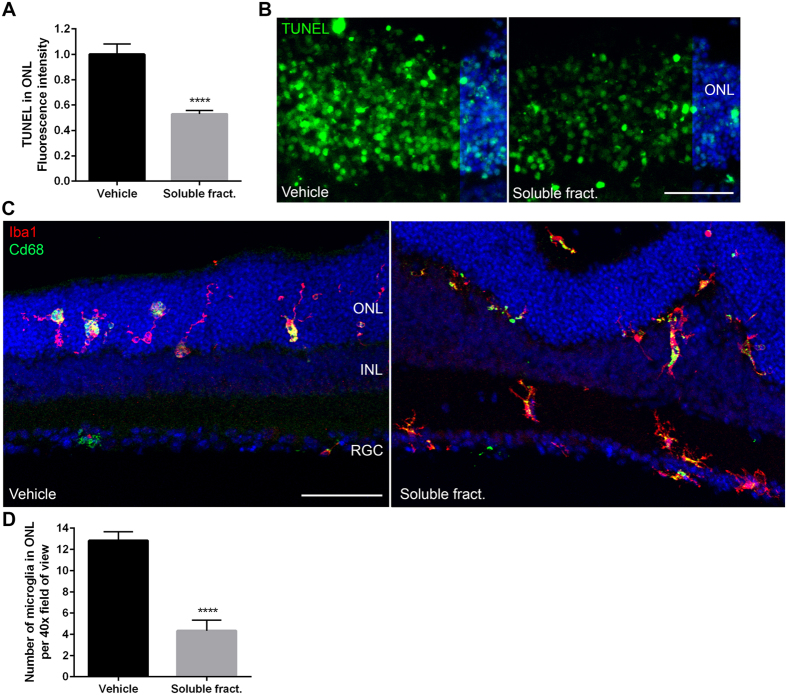
Soluble fractalkine is neuroprotective and modulates microglial migration in rd10 explants. (**A**) Quantification of TUNEL fluorescence intensity in P15 rd10 explants treated with vehicle (0.1% BSA in 1 x PBS) or 100ng/ml recombinant soluble fractalkine (N = 4 explants, n = 4 technical replicates). Scale bar 30 μm. (**B**) Example images of TUNEL reactivity (green) in the ONL of rd10 explants treated with vehicle or soluble fractalkine. (**C**) Example images of microglia (Iba1; red) and activated microglia (CD68; green) in rd10 explants treated with vehicle or recombinant soluble fractalkine. Scale bar 50 μm. (**E**) Quantification of the number of microglia situated in the ONL in rd10 explants treated with vehicle or recombinant soluble fractalkine (N = 4 explants, n = 4 technical replicates). Hoechst reveals cell nuclei. Results are presented as mean ± SEM (t-test, ****p < 0.0001).

Confirmation of a critical role for fractalkine-CX3CR1 signaling in the neuroprotective properties of Norgestrel prompted us to investigate the effects of soluble fractalkine on microglial activity and photoreceptor survival. In support of other studies, we show that administration of recombinant fractalkine to P15 rd10 retinal explants for 24 hr is neuroprotective (Fig. 6A,B)^9^. As expected based on previous observations^25^, microglia were located in close association with clusters of photoreceptors in the rd10 retina at P15 and were positive for phagocytic markers (CD68) (Fig. 6C,D). Following treatment with soluble fractalkine, numbers of microglia in the P15 rd10 ONL decreased significantly (Fig. 6C,D). Microglia appeared to migrate away from the ONL and were mainly observed in the inner nuclear layer (INL) and retinal ganglion cell (RGC) layer (Fig. 6C). These microglia presented a more scavenging, migratory phenotype with less CD68 immunoreactivity, compared to vehicle (Fig. 6C).

**Figure 7:**
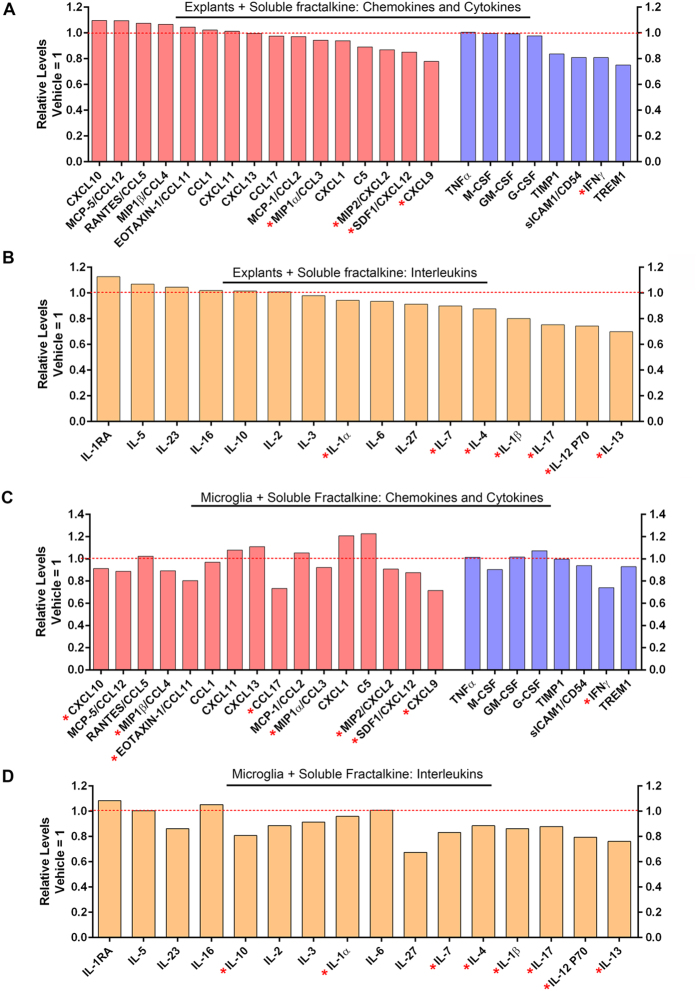
Soluble fractalkine modulates cytokine release in rd10 explants and microglia. (**A**,**B**) Relative levels of (**A**) chemokines and cytokines and (**B**) interleukins in P15 rd10 explants treated with 100 ng/ml soluble fractalkine compared to vehicle. Vehicle (0.1% BSA in 1x PBS) for each cytokine is represented by the dotted red line at 1 (N = 4 explants, n = 2 technical replicates). (**C**,**D**) Relative levels of (**C**) chemokines and cytokines and (**D**) interleukins in isolated rd10 microglia treated with soluble fractalkine compared to vehicle. Vehicle for each cytokine is represented by the dotted red line at 1 (N = 12 retinas, n = 2 technical replicates) (* highlights cytokines implicated in retinal degeneration).

**Figure 8:**
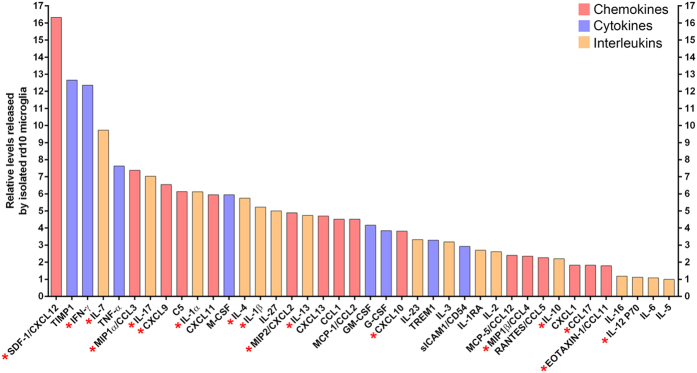
Relative levels of cytokines released by isolated P15 rd10 microglia *in vitro*. Levels of IL-5, the least abundant cytokine detected in media, were set to 1 (N = 12 retinas, n = 2 technical replicates) (* highlights cytokines implicated in retinal degeneration).

Microglia release a variety of pro-inflammatory cytokines, which could contribute to disease progression in the retina^5^,^11^,^27^,^28^,^29^,^30^,^31^. We therefore investigated the effects of fractalkine on cytokine production in rd10 retinal explants and isolated rd10 microglia to further our understanding of the role of fractalkine release in Norgestrel-driven neuroprotection. Using a proteome profiler kit designed to detect a variety of 40 cytokines, we assessed cytokine production in rd10 P15 retinal explants treated with soluble fractalkine for 24 hr. This revealed that cytokine production was altered in rd10 retinal explants as a result of exposure to soluble fractalkine (Fig. 7A,B). As a variety of cell types could be contributing to the reduction in cytokine production in the retina, we assessed cytokine production in isolated rd10 microglia treated with soluble fractalkine. This analysis revealed a similar change in cytokine production in rd10 microglia treated with soluble fractalkine (Fig. 7C,D), highlighting a direct response of microglia to soluble fractalkine in the form of altered cytokine production. Relative levels of the 40 cytokines assessed, released by P15 rd10 microglia *in vitro* (vehicle only), are shown in Fig. 8.

**Figure 9:**
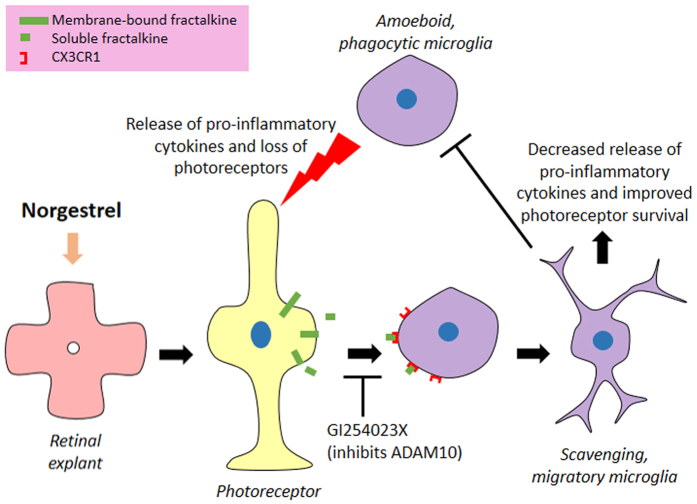
Schematic summarizing the role of fractalkine-CX3CR1 signaling in Norgestrel-mediated retinal neuroprotection. GI254023X is a potent inhibitor of ADAM10, a metalloproteinase responsible for the cleavage of membrane-bound fractalkine.

Amongst the variety of cytokines assessed, we found that several cytokines previously implicated in retinal degeneration were reduced in microglial cultures following treatment with soluble fractalkine (highlighted with * in Figs 7 and 8). This included the following chemokines: MIP1α/CCL3, CXCL10, MIP2/CXCL2, MIP1β/CCL4, eotaxin/CCL11, CCL17 and CXCL9^30^,^32^,^33^,^34^,^35^. Levels of cytokines SDF-1 and IFNγ, also believed to play roles in retinal degeneration^36^,^37^,^38^, were reduced with soluble fractalkine. Amongst the interleukin family, soluble fractalkine treatment resulted in reduced release of IL-1α, IL-4, IL-17, IL-1β, IL-7, IL-10, IL-12 and IL-13 from microglia, all of which have been previously implicated in retinal degeneration^5^,^32^,^39^,^40^,^41^. These results therefore suggest that Norgestrel upregulates the production and release of soluble fractalkine from viable photoreceptors in the diseased retina, which acts on harmful microglia to induce a migratory phenotype and dampen pro-inflammatory responses. The role of fractalkine in Norgestrel’s neuroprotective mechanism is summarized with a schematic in Fig. 9.

## Discussion

In this study, we demonstrate the neuroprotective properties of Norgestrel as a modulator of photoreceptor-microglia crosstalk in the retina. We have previously shown that rd10 microglia drive neuronal cell death of viable 661 W cells *in vitro*, and treating 661 W cells prior to co-culture reduced microglial-driven cell death^7^. In the current study, we wished to investigate this neuroprotective mechanism further, focusing on signaling pathways involved in photoreceptor-microglia crosstalk. Fractalkine-CX3CR1 signaling modulates such crosstalk, and is being considered as a potential molecular target for the treatment of RP^9^. Indeed, we have shown that Norgestrel upregulates fractalkine-CX3CR1 signaling in the rd10 retina 1000 fold at the RNA level, coinciding with significant preservation of the ONL^7^. We therefore designed the current study to investigate the mechanisms underlying Norgestrel-driven, indirect modulation of microglial activity, with a particular focus on the role of fractalkine-CX3CR1 signaling.

We have previously shown that rd10 microglia kill healthy 661 W cells *in vitro*, suggesting that rd10 microglia will drive degeneration of viable photoreceptors *in vivo*^7^. To substantiate this claim, we repeated this co-culture experiment substituting 661 W cells for C57 retinal explants. This confirmed that rd10 microglia drive cell death of viable photoreceptors *ex vivo*, strengthening the hypothesis that rd10 microglia potentiate degeneration in the diseased retina (Fig. 1). Pre-treating 661 W cells and C57 explants with Norgestrel prior to co-culture with rd10 microglia reduced photoreceptor cell death, highlighting the ability of Norgestrel to prime 661 W cells and photoreceptors against potential microglial damage. Potentiation of cell death in the DMSO-treated C57 explant co-cultured with rd10 microglia, coincided with an infiltration and increase of microglia in the ONL (Fig. 2). Microglia were observed along the outer and inner borders of the C57 retinal explant, in contrast to the presence of microglia in the OPL predominantly of the untreated C57 explant. We therefore believe that the source of these infiltrating microglia is the rd10 microglia cultured with the explant.

Norgestrel treatment resulted in increased levels of fractalkine in 661 W cells and C57 retinal explants (Fig. 3). Western blot analyses revealed bands at 100 kDa and 95 kDa, which represent membrane-bound and intra-cellular stores of fractalkine in vesicles respectively^18^,^19^,^20^. A band at 85 kDa reveals levels of soluble fractalkine^19^,^22^. Studies have suggested that premature forms of fractalkine can be observed at 50–70 kDa^18^,^19^. Such bands were observed in the C57 explant but not in 661 W cells. We identified distinct bands at 40 kDa in both 661 W cells and C57 explants. In addition, a prominent band at 30 kDa was observed in C57 explants (Fig. 3H). It is possible that these low molecular weight bands at 30 kDa and 40 kDa, are also premature forms of fractalkine or perhaps products resulting from the cleavage of membrane-bound fractalkine.

Knockdown of fractalkine in 661 W cells and inhibition of fractalkine cleavage in rd10 retinal explants, reduced the protective effects of Norgestrel against microglial damage (Figs 4 and 5). This confirms an essential role for fractalkine-CX3CR1 signaling in the neuroprotection offered by Norgestrel. When fractalkine was knocked down in 661 W cells, Norgestrel’s protective effects against microglia were not completely prevented (Fig. 4; Scrambled vehicle vs siRNA fract. Norg). This is not surprising as fractalkine was reduced rather than absent at the protein and RNA level by siRNA knockdown. Supporting previous observations^9^,^42^,^43^, we have shown in the rd10 retina that soluble fractalkine modulates migration of microglia (Fig. 6). We found that addition of recombinant soluble fractalkine induced a migratory phenotype in rd10 microglia. Indeed, studies have presented similar findings using CX3CR1−/− mice to show that loss of fractalkine signaling reduces motility of both resting and activated microglia in the retina^24^. Studies in the brain have documented an anti-inflammatory role for fractalkine-CX3CR1 signaling^44^,^45^,^46^. Here we reveal a role for soluble fractalkine in dampening pro-inflammatory phenotypes in rd10 retinal microglia (Fig. 7).

In previous studies, we have demonstrated the direct action of Norgestrel on rd10 microglia, dampening pro-inflammatory processes and consequently improving neuronal survival *in vitro*^7^. The current work highlights an additional aspect to Norgestrel’s actions on microglia, acting indirectly through photoreceptors to alleviate harmful microglial activity. In summary, this study highlights a vital aspect to Norgestrel’s neuroprotective properties, alleviating harmful microglial activity through the modulation of photoreceptor-microglia crosstalk. We demonstrate that fractalkine-CX3CR1 signaling plays a critical role in Norgestrel-mediated neuroprotection in the rd10 retina, promoting a migratory phenotype in microglia, downregulating the release of pro-inflammatory cytokines and consequently increasing photoreceptor survival (Fig. 9). These findings reinforce the prospect of Norgestrel as a promising therapeutic for RP.

## Methods

### Mice

All animals were handled and maintained following the Association for Research in Vision and Ophthalmology statement for the Use of Animals in Ophthalmic and Vision Research (License Number AE19130). Experiments were approved by University College Cork Animal Experimentation Ethics Committee and were performed using both male and female homozygous rd10/rd10 mice (B6.CXBI-Pde6b^rd10^/J) and C57BL/6 J mice. Mice were supplied by the Biological Services Unit, University College Cork and were humanely euthanized by cervical dislocation.

### Retinal explant culture

Retinal explants were cultured from P20 C57 and P15 rd10 mice. Eyes were enucleated and transferred to a sterile laminar flow hood. Whole retinas were carefully dissected and placed, photoreceptor side down, on a cell culture insert in R16 medium supplemented with various other compounds^13^. Each explant was cultured in one chamber of a 6-well multi-dish in 1.2 ml of medium with 20 μM Norgestrel (Sigma), 100 ng/ml recombinant mouse soluble fractalkine (R&D systems) or vehicle (DMSO or 0.1% BSA in 1x PBS respectively). For experiments using the ADAM10 inhibitor GI254023X (100 nM, Sigma), explants were pre-treated with inhibitor or vehicle (DMSO) for 1 h before the addition of Norgestrel or vehicle (DMSO).

### Immunohistochemistry on retinal sections

Whole retinal explants were fixed at room temperature in 4% paraformaldehyde (PFA) for 0.5 h. Following washes, retinas were cryo-protected in 15% sucrose in 1xPBS for 1 h, 20% sucrose for 1 h and 30% sucrose overnight, all at 4 °C. Retinas were submerged and frozen in cryochrome (Thermo Scientific, Waltham, US) and sectioned on a cryostat (Leica, Wetzlar, Germany). Sections (7 μm for TUNEL and 20 μm for IF on microglia) were collected on Superfrost glass slides (Fisher Scientific, Waltham, US) and stored at −80 °C. Sections were blocked and permeabilized with 0.1% Triton X and 5% donkey serum in 1xPBS for 30 min and incubated with primary antibody diluted in 5% donkey serum overnight at 4 °C. Table 1 lists the details of all primary antibodies used. Following washes, sections were incubated with secondary antibody (Alexa Fluor donkey anti-rabbit/rat with either a 488 or 594 fluorescent probe; Molecular Probes &) and Hoechst 33342 nuclear stain (1:10,000; ThermoFischer) for 1 h at room temperature. Eliminating the primary antibody in solution served as a negative control (Supplementary Figure S1). Sections were mounted using Mowiol (Sigma) with Dabco anti-fade agent (Sigma).

### Immunohistochemistry on cells

Microglia and/or 661 W cells cultured on polylysine-coated coverslips in 24 well plates were fixed with 4% PFA for 10 min at room temperature. Cells were blocked and permeabilized in 0.1% Triton X and 5% donkey serum in 1xPBS for 10 min and incubated with primary antibody diluted in 5% donkey serum overnight at 4 °C. Table 1 lists the details of all primary antibodies used. Following washes, cells were incubated with secondary antibody (Alexa Fluor donkey anti-rabbit with either a 488 or 594 fluorescent probe) and Hoechst 33342 nuclear stain (1:10,000) for 1 h at room temperature. Eliminating the primary antibody in solution served as a negative control. Cells on coverslips were mounted on to glass slides using Mowiol with Dabco anti-fade agent.

### Microscopy and q**u**antification

Retinal sections and cell preparations were viewed using a Leica DM LB2 microscope with Nikon Digital Sight DS-U2 camera, using a 40x objective. Images were taken using the software NIS-Elements version 3.0, Nikon, Japan. Immunofluorescence on retinal sections was performed on at least three explants of each group. Images were taken and quantification was performed in the central portion of the retina. Immunofluorescence on cell preparations was also performed in triplicate. ImageJ software was used for quantification. Number of microglia contacting 661 W cells included any microglial cell nucleus overlapping with 661 W cell cytoplasm, identified with cone arrestin. Fluorescence intensity measurements of fractalkine in 661 W cells and C57 explants was performed using ImageJ software as previously described^25^. An identical size box was drawn within the boundaries of each cell or within the ONL and used for measurement of average fluorescence intensity. Confocal micrographs were taken using an Olympus Fluoview FV1000 laser scanning confocal microscope, using a 20x objective. Images were taken using the software Olympus Fluoview Ver 4.1a and are represented as maximum intensity projections from acquisition of z-stacks. Identical microscope settings were used when visualizing specific markers across treatments.

### Terminal dUTP Nick-End Labeling (TUNEL) of fragmented DNA

DNA strand breaks in retinal explant sections and 661 W cells were detected by terminal dUTP nick end-labeling (TUNEL) on fixed tissue and cells. Retinal sections or cells were permeabilized with 0.1% Triton X for 2 min followed by incubation with terminal deoxynucleotidyl transferase (Promega, Wisconsin, US) and fluorescein-12-dUTP (Roche, Risch-Rotkreuz, Switzerland) according to manufacturer’s instructions. Nuclei were counterstained with Hoechst 33342 (1:10,000) (Sigma). Sections and cells were incubated at 37 °C for 1 h in a humidified chamber and following several washes in 1xPBS, were mounted in Mowiol. Sections were viewed under a fluorescence microscope (Leica DM LB2). Eliminating the TdT enzyme served as a negative control (Supplementary Figure S1). In co-culture assays, TUNEL-positive 661 W cells could easily be distinguished from microglia based on nuclear size and phase contrast of cell preparations. Fluorescence intensity measurements of TUNEL in the ONL was performed using ImageJ software. An identical size box was drawn within the boundaries of the ONL for each explant and used to take five independent measurements, to be averaged.

### Culture of cell lines

Experiments were carried out using the mouse 661 W cone photoreceptor-derived cell line (passage 25–35), generously provided by Dr Muayyad Al-Ubaidi (Department of Cell Biology, University of Oklahoma, Health Sciences Centre, Oklahoma City, OK, USA). This cell line was previously validated by this group through real time quantitative polymerase chain reaction (rt-qPCR) analysis for cone specific opsins; blue cone opsin (Opn1sw) and red/green opsin (Opn1mw)^17^. Cells were cultured in Dulbecco’s Modified Eagle’s medium (DMEM) (Sigma) supplemented with 10% fetal calf serum (FCS) and 1% penicillin streptomycin (PS) and maintained at 37 °C in a humidified 5% CO_2_ atmosphere. To analyze the effects of Norgestrel (Sigma) on 661 W cells, cells were seeded in to the appropriate culture vessel and allowed to attach overnight. Cells were washed with warmed 1xPBS and complete medium supplemented with 20 μM Norgestrel or vehicle (DMSO) was added.

### Western Blotting

661 W cells were detached using accutase and centrifuged at 1,000 rpm for 5 min. 661 W cell pellets and whole retinas were homogenized in RIPA buffer (Thermo) containing protease inhibitors (Thermo). Lysates were centrifuged at 10,000 rpm at 4 °C for 30 min. Supernatant was stored at −80 °C. Protein concentration was measured using a Bradford assay (Bio-Rad, Hercules, US). 4–15% gradient gels (Bio-Rad) were used for SDS-PAGE and proteins were then transferred to a nitrocellulose membrane. 30 μg of protein was loaded per sample. Total protein levels are an accurate way of verifying equal loading^47^ and so were analyzed using REVERT total protein stain (LiCor, Lincoln, US) as per manufacturer’s instructions and imaged on a LiCor scanner in the 700 channel. Membranes were blocked using Odyssey blocking buffer (LiCor) for 30 min and probed with fractalkine primary antibody (1:1000, Abcam, Cambridge, UK) or CX3CR1 (1:500, Abcam) in blocking buffer and 0.1% Tween 20 for 4 nights at 4 °C. Membranes were washed using TBS-T and probed with rabbit 800 secondary antibody (LiCor). Membranes were imaged on a LiCor scanner. Blots are representative of three biological replicates.

### Culture of primary microglial cells

A protocol for isolating and culturing retinal microglia was adapted from a previously published protocol^48^ as previously described^7^. Culture purity was confirmed by immunofluorescence^7^. Briefly, retinas were dissected from the eyes of P15 rd10 mice, ensuring minimum contamination with vitreous body and retinal pigment epithelium. Six retinas were pooled, cut into small pieces and incubated for 40 min at 37 °C in 1 ml 1xPBS with 1 mg/ml collagenase type I (Sigma), 0.3 mg/ml DNase I (Roche, Basel, Switzerland), and 0.2 mg/ml hyaluronidase (Sigma). The cell suspension was filtered through a 70-μm cell strainer (Becton Dickinson, Franklin Lakes, US). Cells were washed twice with 10 ml DMEM/10% FCS/1% PS and suspended in 15 ml DMEM/10% FCS/1% PS. To isolate mononuclear cells, the suspension was gently added to 15 ml Ficoll paque premium reagent (GE Life Sciences, Buckinghamshire, UK) and centrifuged for 20 min at 2,000 rpm without the brake in a Beckmann GS-6R centrifuge. The interphase was removed carefully and washed twice with 10 ml DMEM/10% FCS/1% PS. Primary microglia were cultured on polylysine-coated coverslips in 24 well plates for 1 day before the addition of 661 W cells, or prior to treating with recombinant mouse soluble fractalkine (100 ng/ml, R&D systems) or vehicle (0.1% BSA in 1xPBS) for 24 h. Conditioned media was collected and centrifuged at 1,000 rpm for 5 min. Supernatant was stored at −80 °C.

### Co-culture of rd10 microglia with 661 W cells

Primary microglia were cultured on polylysine-coated coverslips in 24 well plates for 1 day, and washed with 1xPBS prior to the addition of healthy 661 W cells that had been treated with Norgestrel or vehicle (DMSO) in the preceding 24 h. 661 W cells suspended in fresh DMEM/10% FCS/1% PS were added to the microglia at a density of 20 × 10^3^ per well. Co-cultures of microglia and 661 W cells were left for 24 h before fixation in 4% PFA.

### Co-culture of rd10 microglia with C57 explants

C57 P20 retinal explants were cultured in the presence of 20 μM Norgestrel or vehicle for 5 hr and washed with 1xPBS prior to the addition of isolated rd10 P15 retinal microglia. Rd10 microglia isolated as described in previous sections were cultured with explants for a further 19 hr.

### Assessment of cell viability in 661 W cells by the MTS assay

Cells seeded overnight (2 × 10^3^ per well 96 well plate) were washed three times in 1xPBS before treatment. After 20 h of treatment incubation, 20 μl of MTS solution (Promega) was added to each well and incubated for a further 4 h at 37 °C. Viable cells in the presence of phenazine methosulfate (PMS) will reduce the MTS solution to form formazan. Detection and quantification of the formazan crystals is then carried out with a microplate reader (Molecular Device Corporation, Sunnyvale, US) at 490 nm. 490 nm readings taken from non-template wells (media and MTS, without cells) were deducted from actual cellular readings. A further reading at 650 nm was also taken from all wells and deducted from the 490 nm readings to account for any cellular debris. The quantity of formazan product as measured by the amount of 490 nm absorbance is directly proportional to the number of living cells in culture. Therefore the absorbance of the formazan formed in ‘control cell wells’ was taken as 100% viability.

### Total RNA Isolation and quantitative real time polymerase chain reaction (rt-qPCR)

Total RNA was isolated from whole 661 W cells using RNeasy Mini Kit (Qiagen) following manufacturer’s protocol. Genomic DNA was eliminated in 661 W cells using RNase free DNase (Qiagen) and cDNA was subsequently synthesized using QuantiTect Reverse Transcription Kit (Qiagen). Rt-qPCR was performed using QuantiTect SYBR Green PCR Kit (Qiagen) and 10 ng cDNA/well of a 384 well plate (Starstedt AG & Co.) for 661 W cells. Plates were run using the Applied Biosystems 7900HT Fast Real-Time PCR System (Life Technologies Ltd., Carlsbad, US) and each set of reactions included both a non-reverse transcription control and a no template sample negative control. The protocol consisted of a cycling profile of 30 s at 95 °C, 60 s at 60 °C, and 30 s at 72 °C for 40 cycles. Qiagen QuantiTect primer assays for fractalkine (QT00128345), actin (QT0095242), GAPDH (QT0165692) and HPRT (QT00166768) were used. Melt curve analysis confirmed a single PCR product. Relative changes in gene expression were quantified using the comparative Ct (ΔΔCt) method as described by Livak & Schmittgen^49^,^50^. The Ct value of the gene of interest was normalized to an average of three endogenous housekeeping genes (Actb, Gapdh and Hprt). This was compared to the normalized control sample. Alteration in mRNA expression of genes was defined as fold difference in the expression level in cells after treatment, relative to that of the control. This is the standard method for presenting rt-qPCR data^49^,^50^.

### siRNA transfection in 661 W cells

Cells were seeded 6 h prior to transfection. Fractalkine GeneSolution siRNA (5 nM, Qiagen) and Allstars negative control siRNA (5 nM, Qiagen) were then transfected in to the cells using HiPerFect transfection reagent (Qiagen). Cells were transfected for 72 hr before treatment with Norgestrel or vehicle for a further 24 hr.

### Cytokine proteome profiler array

Rd10 P15 retinal explants and isolated microglia were treated with soluble fractalkine (100 ng/ml) or vehicle (0.1% BSA in 1xPBS) for 24 hr. Explants were homogenized in RIPA buffer (Thermo) containing protease inhibitors (Thermo). Lysates were centrifuged at 10,000 rpm at 4 °C for 30 min and supernatants stored at −80 °C. Protein concentration was calculated using the Bradford assay (Biorad). A single lysate solution for each treatment (vehicle or soluble fractalkine) contained 300 μg of protein from three explants, using 100 μg of protein from each explant. For primary microglia, conditioned media from vehicle or soluble fractalkine-treated cells was collected. Twelve retinas from six rd10 mice age P15 were used to make the primary culture. Media was centrifuged at 1,000 rpm at 4 °C for 5 min to eliminate debris. The proteome profiler mouse cytokine array panel A kit (R&D systems) was used to assess the concentration of 40 cytokines in samples of interest. As per manufacturers’ instructions, a single membrane with antibodies against 40 cytokines in duplicate, was used per treatment group. Membranes were blocked with the supplied blocking buffer and incubated with a solution of explant lysate or microglial conditioned media overnight at 4 °C. Following washes, membranes were incubated with IRDye 800CW Streptavidin (1:2,000, LiCor) in blocking buffer for 30 min. Membranes were washed and imaged on a LiCor scanner in the 800 channel. Fluorescence intensity was measured using Image Studio Lite software. The average intensity of the two duplicate spots per antibody was calculated. Vehicle values of each cytokine were set to 1 and the resulting relative values for the treatment group plotted on a bar graph.

### Statistical analysis

Values in all graphs represent the mean ± standard error of the mean (SEM) and are representative of at least three replicates. Data were statistically analyzed using Student t-test or two-way ANOVA with multiple comparisons (Graph Pad, Prism 6) with values of *p* < 0.05 being considered statistically significant.

## Additional Information

**How to cite this article**: Roche, S. L. *et al*. Fractalkine-CX3CR1 signaling is critical for progesterone-mediated neuroprotection in the retina. *Sci. Rep.* **7**, 43067; doi: 10.1038/srep43067 (2017).

**Publisher's note:** Springer Nature remains neutral with regard to jurisdictional claims in published maps and institutional affiliations.

## Supplementary Material

Supplementary Figure S1

## Figures and Tables

**Table 1:** List of antibodies used for Western blotting and immunofluorescence.

Antibody	Supplier	Catalogue #	Host	Dilution Factor
Iba1	Wako	019–19741	Rabbit polyclonal	IF 1:500
CD68	AbD Serotec	MCA1957GA	Rat monoclonal	IF 1:500
Cone Arrestin	Millipore	AB15282	Rabbit polyclonal	IF 1:1,000
Fractalkine	Abcam	AB25088	Rabbit polyclonal	IF: 1:1,000 WB 1:1,000
CX3CR1	Abcam	AB8021	Rabbit polyclonal	WB 1:500
