# Supplementary material for: Fractalkine-CX3CR1 signaling is critical for progesterone-mediated neuroprotection in the retina
Source: Sci Rep. 2017 Feb 20;7:43067. doi: 10.1038/srep43067 (PMC5316933; doi:10.1038/srep43067)
Supplement: Supplementary Figure S1 [file srep43067-s1.pdf]

Supplementary Files

Fractalkine-CX3CR1 signaling is critical for progesterone-mediated neuroprotection in the retina

Sarah L Roche[1], Alice C Wyse-Jackson[1], Ana M Ruiz-Lopez[1], Ashleigh M Byrne[1] and Thomas G Cotter[1]*

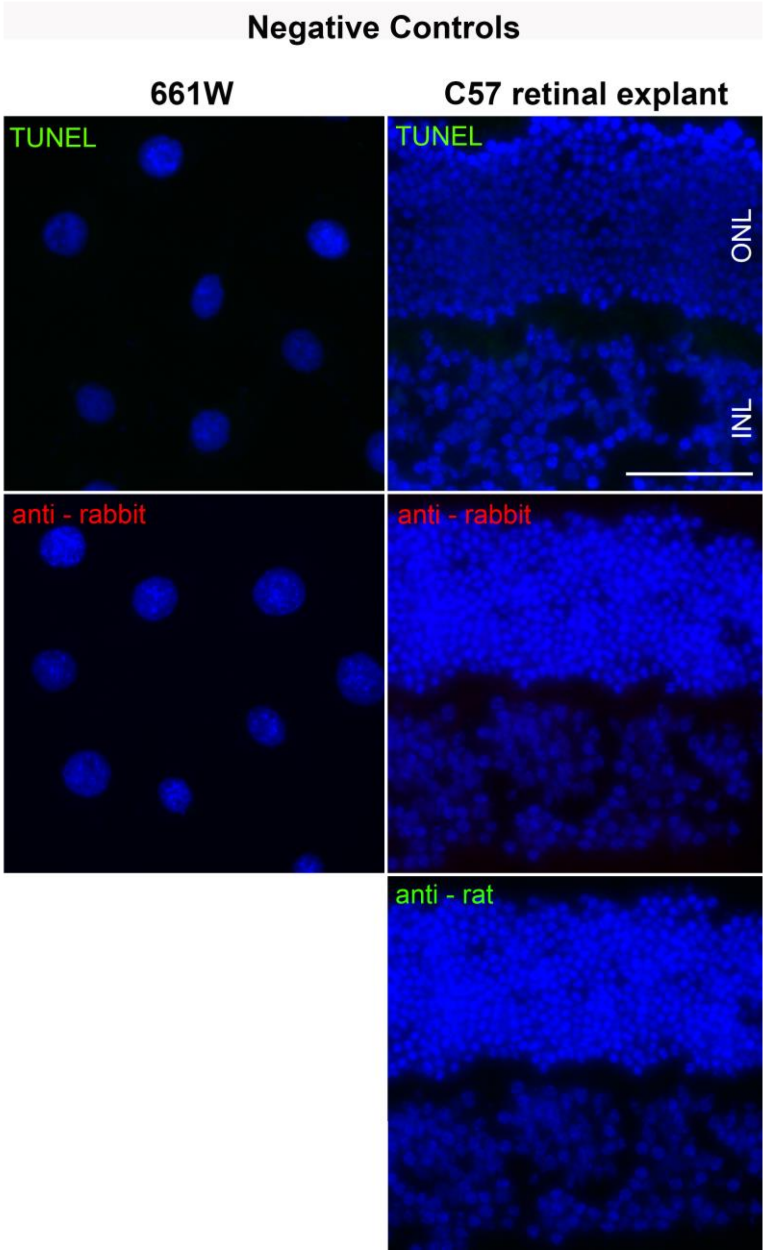

Supplementary Figure S1. Example images of negative controls for TUNEL (elimination of TdT enzyme) and immunofluorescence (elimination of secondary antibody) in 661W cells and C57 retinal explants. Scale bar 50µm.
